# Supplementary material for: An Approach to Intersectionally Target Mature Enteroendocrine Cells in the Small Intestine of Mice
Source: Cells. 2024 Jan 4;13(1):102. doi: 10.3390/cells13010102 (PMC10778503; doi:10.3390/cells13010102)
Supplement: Supplementary file 1 [file cells-13-00102-s001.zip › cells-2769437-supplementary.pdf]

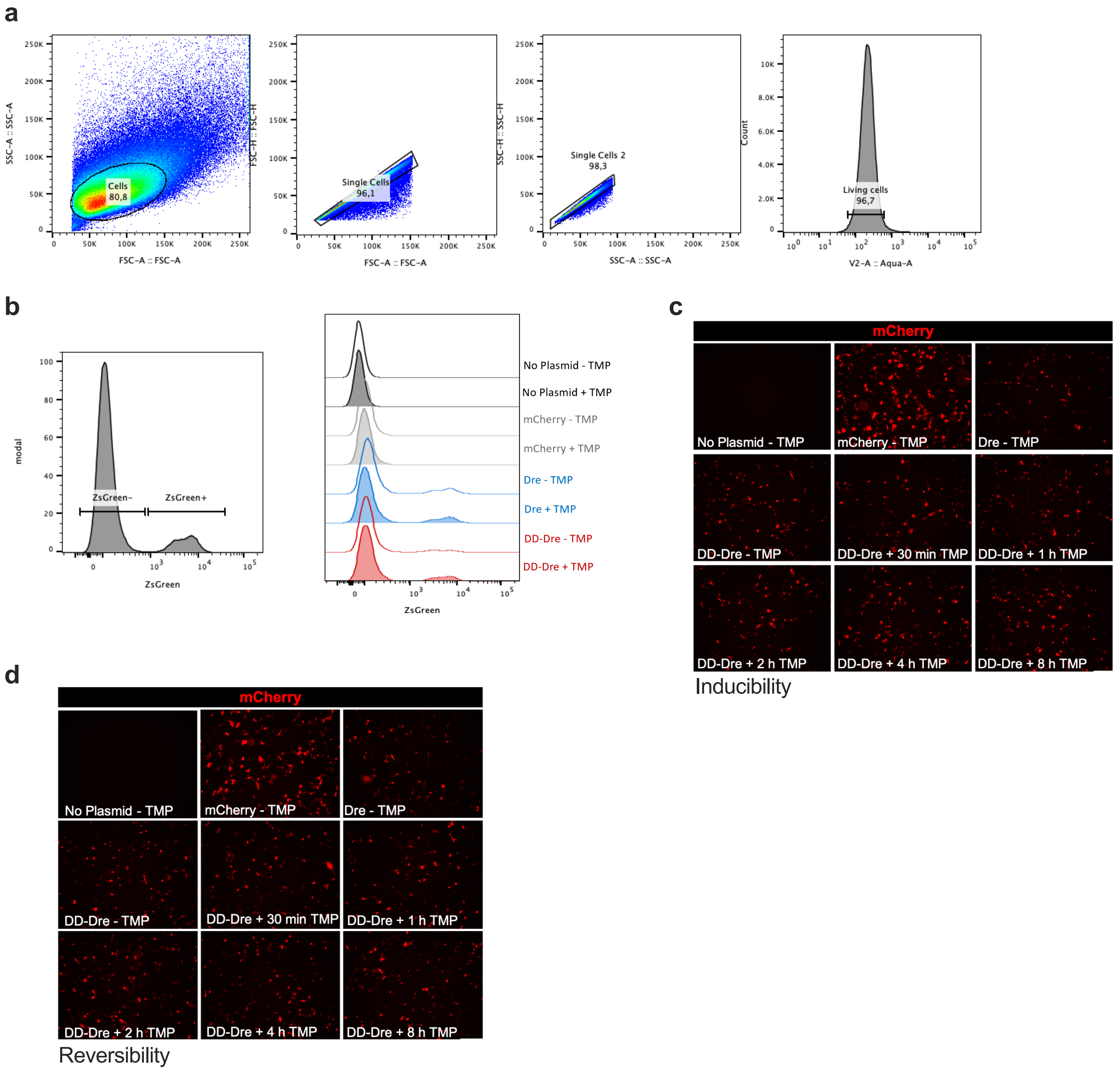

Figure S1: Assessment of a TMP-inducible Dre system *in vitro*

a) Representative FACS gating strategy for Figure 1b. b) Representative plot of the total cell population sorted by ZsGreen fluorescence and split by condition. c) Representative mCherry fluorescence microscopy images as control for a uniform transfection in each sample for Figure 1 C, D. Cells were transfected with pTEDre/pTE-DD-Dre/pCAGGS-mCherry plasmids and treated up to 8 h with TMP. Images were taken 24 h post transfection. Scale bar: 250 µm. d) Representative mCherry fluorescence microscopy images as control for a uniform transfection in each sample for Figure 1 E, F. TMP washout was monitored for 8 h. Images were taken 24 h post transfection. Scale bars: 250 µm.

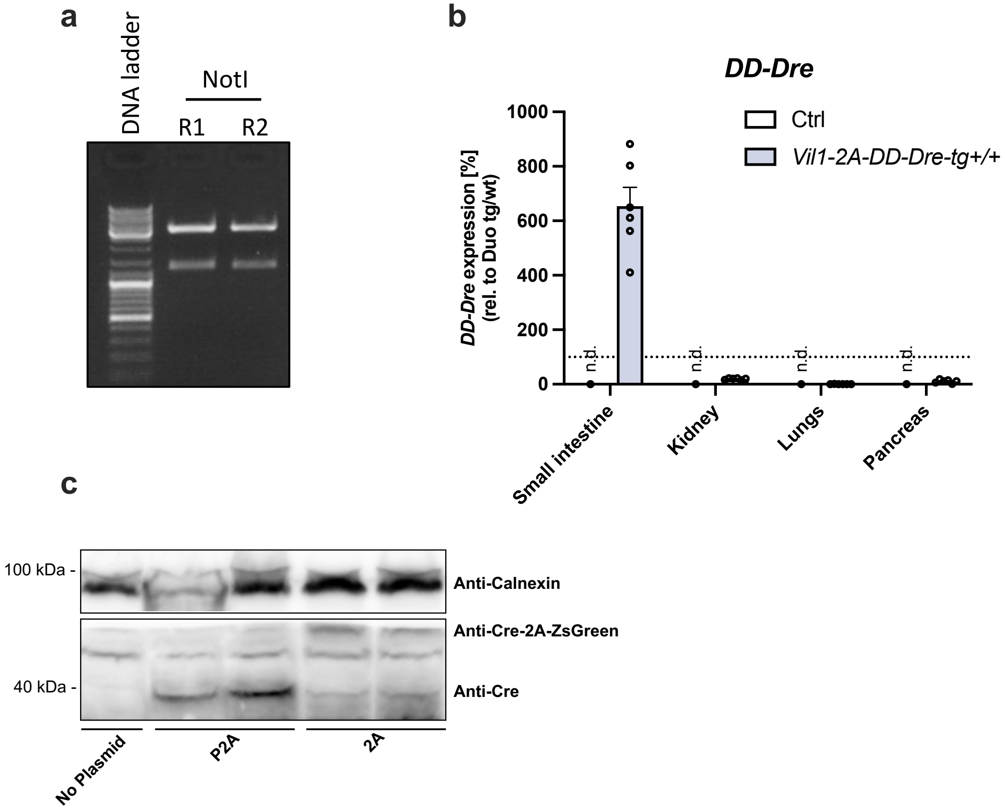

Figure S2: a) Control digest of PCR products of R1 and R2 after successful subcloning into pGEM-T easy using NotI. b) *DD-Dre* expression was measured by qPCR across organs derived from indicated mice. Data were normalized to *DD-Dre* expression in duodenum from *Vil1-2A-DD-Dre-tg+/-* mice shown in Figure 2d. c) Western blot analysis using anti-Cre and anti-Calnexin (loading control) antibodies of MEFs transiently transfected w/o plasmid, with *Cre-2A-ZsGreen* and *Cre-P2A-ZsGreen* encoding plasmids.

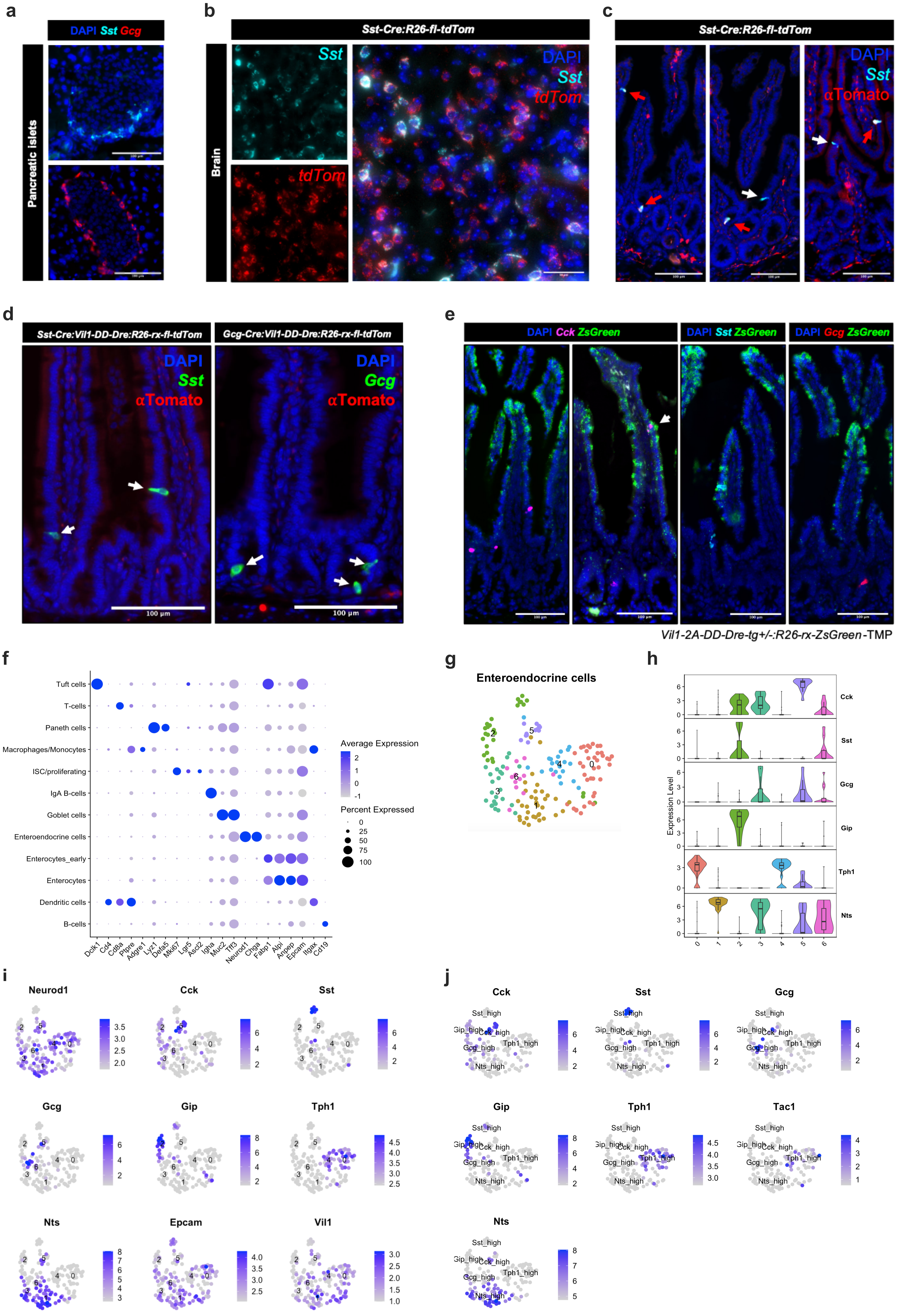

a
DAPI Sst Gcg
Pancreatic islets
b
Sst-Cre:R26-fl-tdTom
Brain
Sst
tdTom
DAPI
Sst
tdTom
c
Sst-Cre:R26-fl-tdTom
DAPI
Sst
αTomato
d
Sst-Cre:Vil1-DD-Dre:R26-rx-fl-tdTom
Gcg-Cre:Vil1-DD-Dre:R26-rx-fl-tdTom
DAPI
Sst
αTomato
DAPI
Gcg
αTomato
100 µm
100 µm
e
DAPI Cck ZsGreen
DAPI Sst ZsGreen
DAPI Gcg ZsGreen
Vil1-2A-DD-Dre-tg+/-:R26-rx-ZsGreen-TMP
f
Tuft cells
T-cells
Paneth cells
Macrophages/Monocytes
ISC/proliferating
IgA B-cells
Goblet cells
Enteroendocrine cells
Enterocytes_early
Enterocytes
Dendritic cells
B-cells
Dclk1
Cd4
Cd8a
Ptprc
Adgre1
Lyz1
Defa5
Mki67
Lgr5
Ascl2
Igha
Muc2
Tff3
Neurod1
Chga
Fabp1
Alpi
Anpep
Epcam
Itgax
Cd19
Average Expression
2
1
0
-1
Percent Expressed
0
25
50
75
100
g
Enteroendocrine cells
h
Cck
Sst
Gcg
Gip
Tph1
Nts
Expression Level
i
Neurod1
Cck
Sst
Gcg
Gip
Tph1
Nts
Epcam
Vil1
j
Cck
Sst
Gcg
Gip
Tph1
Tac1
Nts
Sst_high
Gip_high
Cck_high
Gcg_high
Tph1_high
Nts_high

Figure S3: An intersectional approach to target mature EEC populations

a) RNAScope ISH of pancreatic islets against *Sst* or *Gcg* counterstained with DAPI. b) RNAScope ISH of brain sections against *Sst* and *tdtomato* counterstained with DAPI of indicated mice. Scale bar: 50 µm. c) Anti-tdTomato IHC combined with RNAScope ISH against *Sst* of small intestines from indicated mice. Red arrows indicate *Sst*/tdTom double-positive cells. White arrows indicate *Sst*-only positive cells. d) Anti-tdTomato IHC combined with RNAScope ISH against *Sst* or *Gcg* counterstained with DAPI of small intestines from indicated mice. White arrows indicate *Sst*$^{+}$/*Gcg*$^{+}$ and tdTomato^{-} EECs. e) *Cck/Sst/Gcg* and *ZsGreen* multiplex RNAScope of *Vil1-2A-DD-Dre-tg+/-:R26-rx-ZsGreen* mice without TMP. White arrow indicates co-expression of *Cck* and *ZsGreen.* Scale bars in a, c, d and e represent 100 µm. f) Gene expression markers that were used for manual annotation of UMAP clusters in Figure 4e across the annotated celltypes. g) Isolated and re-clustered EEC population. 7 clusters were found. However, due to low cell numbers unsupervised clustering was not sufficient to separate EEC subpopulations. h) EEC subtype marker expression across the 7 EEC clusters. i) EEC subtype and IEC marker expression across the 7 EEC clusters. *Vil1* is mostly expressed by cluster 0 and 1. j) EECs subtype marker expression across the annotated EEC subpopulations. Cells were annotated based on threshold expression of the indicated genes.
